# Spider Communities and Biological Control in Native Habitats Surrounding Greenhouses

**DOI:** 10.3390/insects9010033

**Published:** 2018-03-14

**Authors:** Belén Cotes, Mónica González, Emilio Benítez, Eva De Mas, Gemma Clemente-Orta, Mercedes Campos, Estefanía Rodríguez

**Affiliations:** 1 Department of Plant Protection Biology, Swedish University of Agricultural Sciences, 23053 Alnarp, Sweden; belen.ramal@plen.ku.dk; 2 Estación Experimental Cajamar, El Ejido, 04710 Almeria, Spain; monicagonzalez@fundacioncajamar.com (M.G.); gemma.clemente@pvcf.udl.cat (G.C.-O.); 3 Department of Environmental Protection, Estación Experimental del Zaidín, CSIC, 18008 Granada, Spain; emilio.benitez@eez.csic.es (E.B.); mercedes.campos@eez.csic.es (M.C.); 4 Department of Functional and Evolutionary Ecology, Estación Experimental de Zonas Aridas, CSIC, 04120 Almeria, Spain; evademas@gmail.com; 5 IFAPA, Centro La Mojonera, 04745 Almeria, Spain

**Keywords:** beneficial arthropods, GAM, habitat manipulation, RDA, tobacco whitefly, western flower thrips

## Abstract

The promotion of native vegetation as a habitat for natural enemies, which could increase their abundance and fitness, is especially useful in highly simplified settings such as Mediterranean greenhouse landscapes. Spiders as generalist predators may also be involved in intra-guild predation. However, the niche complementarity provided by spiders as a group means that increased spider diversity may facilitate complementary control actions. In this study, the interactions between spiders, the two major horticultural pests, *Bemisia tabaci* and *Frankliniella occidentalis*, and their naturally occurring predators and parasitoids were evaluated in a mix of 21 newly planted shrubs selected for habitat management in a highly disturbed horticultural system. The effects of all factors were evaluated using redundancy analysis (RDA) and the generalized additive model (GAM) to assess the statistical significance of abundance of spiders and pests. The GAM showed that the abundance of both pests had a significant effect on hunter spider’s abundance, whereas the abundance of *B. tabaci*, but not *F. occidentalis*, affected web-weavers’ abundance. Ordination analysis showed that spider abundance closely correlated with that of *B. tabaci* but not with that of *F. occidentalis*, suggesting that complementarity occurs, and thereby probability of biocontrol, with respect to the targeted pest *B. tabaci*, although the temporal patterns of the spiders differed from those of *F. occidentalis*. Conservation strategies involving the establishment of these native plants around greenhouses could be an effective way to reduce pest populations outdoors.

## 1. Introduction

Biodiversity in agro-ecosystems can enhance ecosystem pest control, which can potentially reduce the reliance on chemical inputs such as pesticides [1,2,3]. Perennial non-crop habitats are thought to play a crucial role in maintaining the natural enemies (NEs) of pest populations in agricultural landscapes. Semi-natural habitats can provide shelter, floral food resources (nectar and/or pollen), alternative prey and hosts [4,5]. However, various studies have recorded neutral, positive and negative relationships between NEs biodiversity and the resulting effectiveness of biological control (BC), caused by functional redundancy, niche complementarity and intra-guild predation (IGP), respectively [6,7]. Successful management of this ecosystem service therefore depends on the development of appropriate plant diversity rather than an increase in the number of plant species per se [4,5].

In conventional agriculture systems, which are highly dependent on synthetic inputs, biodiversity is usually limited, which, in turn, limits the ability to provide BC services [1,2,3,8]. Intensive greenhouse horticulture is located in the most arid regions of the Mediterranean Basin. The province of Almeria (SE Spain) is the most dominant horticultural region in Europe encompassing 30,000 ha of plastic greenhouses [9]. Economic development in this region has been prioritized over long-term environmental issues, which has resulted in the loss of native perennial vegetation and considerable fragmentation [10]. 

The tobacco whitefly, *Bemisia tabaci* (Gennadius) (Homoptera: Aleyrodidae), and the western flower thrips, *Frankliniella occidentalis* (Pergande) (Thysanoptera: Thripidae), are the insect pest species most commonly found in this horticultural system. These are, by far, the most problematic pests, given their polyphagous habits and their capacity to transmit a large number of plant viruses [11]. Integrated Pest Management (IPM) practices have been adopted by a majority of growers since 2008, and use of effective native NEs plays an important role in pest control [12,13,14,15]. According to local government data, biological control plays a vital role in about 80% of the greenhouse crops in Almeria. In this less pesticide-dependent context, some strategies have been proposed. For example, hedgerows containing native shrubs can be established between greenhouses to promote biodiversity and ecosystem services [16,17]. In fact, it is known that native flora may act as suitable habitat for beneficial insects in agroecosystems [18,19,20,21]. In this sense, authors found that native shrubs surrounding greenhouses in Almeria are unsuitable as reservoirs for plant virus [17]. In addition, some native shrubs have also been identified as hosts of the two main pests and their specifics NEs [22]. However, the function of these native shrubs as suitable habitat for other important predators like spiders remains unknown.

As a major predator group, spiders (Araneae), which feed on terrestrial arthropod communities, are one of the most abundant, diversified and ubiquitous populations in both natural and agricultural habitats [23,24]. Spiders, whose role as BC agents in agroecosystems have been well documented, can also have significant top-down effects [25,26,27]. Spider assemblages can offer a complementary niche to attack different pest species or subsets of the same pest species [28]. Similarly, their predator foraging behaviour has a variety of indirect effects on other species and, ultimately, on plant communities [29]. The current status of spiders as generalist predators could limit their biocontrol potential due to their involvement in IGP [30,31]. Therefore, apart from the potential for intra-guild predation, a diversified assemblage of spiders may exert a natural biological control. Some studies have shown that whiteflies and thrips make up a spider’s diet in some crops [32,33,34,35,36,37]. Data on spider diversity in arid areas of SE Spain indicate that spider assemblages are highly diverse [38]. However, the impact of spider communities existing in and interacting with native shrubs surrounding greenhouses is unknown. Spiders may represent potential biological agents for the main horticultural pests, or maybe a risk to the conservation of key NEs. Therefore, the aim of this study was (1) to identify native plants that support spider guilds and (2) to assess the specific relationships between spider guilds, pests and other NEs. This is a necessary first step for future research based on selection of plants for increasing biocontrol services by spiders outside the greenhouses in order to reduce the pest population and decrease their ability to colonize the greenhouses.

## 2. Materials and Methods

### 2.1. Study Area and Plant Species

Field surveys were conducted at Cajamar Experimental Station in the province of Almeria, Spain, at 36°48′ N, 2°3′ W and at an elevation of ~155 m. The experimental field was divided into 4 plots (17 × 10 m) separated by four walkways with a width of 1–2 m that were kept bare [22]. In December 2010, we established a semi-arid shrubland patch with a pool of 21 plant species approximately three years old in each plot and belonging to 12 different botanical families. Each plant species was replicated in a different ratio according to its size (Table 1). The design aims to reproduce the natural environment. The ratio was approximately: shrubs at 1 m (ratio 1:1), small shrubs at 0.5 m (ratio 2:1) and ground cover species at 0.30 m (ratio 3:1). This allows for creating a range of vegetation strata. The experimental design included nectar-rich plants (8 species), pollen-rich plants (6 species) and pollen–nectar-rich plants (7 species). For the purposes of plant selection, other criteria were also considered using a ranking system based on the following criteria [16]: (1) overlapping bloom periods, (2) non-hosting horticultural virus diseases [17], (3) as well as morphology and colour. All plots were manually weeded during the study. The field was located in an area completely surrounded by greenhouses in the size range of 1000–2000 m^2^, and with different vegetable production including: tomato, pepper and cucumber. The neighbouring horticultural crops were managed under an integrated pest management regime with an emphasis on augmentative BC. The NEs, which are mass-reared and widely commercialized in the study area, included: the whitefly parasitoid *Eretmocerus* spp. (Hymenoptera: Aphelinidae), the whitefly predator *Nesidiocoris tenuis* (Reuter) (Hemiptera: Miridae), the aphid parasitoid *Aphidius colemani*, as well as the thrips predators *Orius laevigatus* (Hemiptera: Anthocoridae) and the predatory mite *Amblyseius swirskii* (Acari: Phytoseiidae).

### 2.2. Arthropod Collection

After 18 months, when the native plants were well established, the sampling of arthropods was carried out in 161 plants once a month between June 2012 and June 2013 [22]. Arthropods from each plant were vacuumed for 40 s with the aid of a Stihl^®^ SH 85C blower [18]. Among the diverse range of arthropods collected, the primary focus was on spiders, the two horticultural pests *B. tabaci* and *F. occidentalis* and their naturally occurring NEs: *N. tenuis*, *O. laevigatus*, *Eretmocerus* spp*.*, the thrips parasitoid *Ceranisus* spp. (Hymenoptera: Eulophidae) and the thrips predator *Aeolothrips* spp*.* (Thysanoptera: Aeolothripidae). Juvenile spiders were taxonomically sorted by family, the adults by species, and, where possible, by morphospecies. They were also sorted by functional group into web-weaver and hunter spiders (the latter involved in non-web foraging) [35].

### 2.3. Statistical Analysis

Data analysis began with data exploration [39]. Boxplots were used to show the abundance of the different arthropod groups in each plant species. A scatterplot of spider abundance clearly showed a non-linear temporal pattern; non-linear patterns were also found in the residuals when linear regression was applied. Total abundance of spiders was modelled as a function of four covariates by fitting a generalized additive model (GAM) with a Poisson distribution and the logistic link function log(π*_i_*) = η*_i_*. The GAM can handle a combination of parametric and nonparametric variables [40], allowing for non-linear relationships between the response variables and all or some explanatory variables. Two separate GAMs were constructed with abundance of hunter and web-weaver spiders as response variables. The covariates used in the predictor function (η*_i_*) were a group of twenty-one plant species, whitefly abundance, thrips abundance and smoothing function of time (sampling month). Thus, the Poisson GAM for hunter spiders is specified below:

*Hunters_i_* ~ *Poisson*(µ_*i*_) (1)

*E*(*Hunters_i_*) = var(*Hunters_i_*) = µ_*i*_ (2)

*log*(µ_*i*_) = *α* + *β*_1_ × *Plant species_i_* + *β*_2_ × *Whitefly abundance_i_* + *β*_3_ × *Thrips abundance_i_* + *f*(*Time_i_*) (3)

The Poisson GAM for web-weaver spiders is given below:

*Web-weavers_i_* ~ *Poisson*(µ_*i*_) (4)

*E*(*Web-weavers_i_*) = var(*Web-weavers_i_*) = µ_*i*_ (5)

*log*(µ_*i*_) = *α* + *β*_1_ × *Plant species_i_* + *β*_2_ × *Whitefly abundance_i_* + *β*_3_ × *Thrips abundance_i_* + *f*(*Time_i_*) (6)

The models were built using R software [41] and were implemented with the aid of the mgvc package [42]. Overdispersion, which was determined by the sum of the squared Pearson residuals, divided by the residual degree of freedom, was found to be less than 2 in all models presented.

To graphically represent multivariate arthropod composition patterns, the redundancy analysis (RDA) ordination technique was used to determine the relationships between the three arthropod groups (insect pests, NEs and spiders) and explanatory variables (plant species and sampling month). The results are displayed graphically in an RDA correlation triplot using the vegan package [42].

## 3. Results

### 3.1. Spider Composition in Native Plants

A total of 1301 spiders were collected during the sampling period. While five families of web-weavers were collected throughout the study, the species, *Neoscona subfusca*, constituted the most abundant spider in native plants. Hunters were composed of six families, with Salticidae, Thomisidae and Philodromidae predominating among the captures (Table 2).

**Figure 1:**
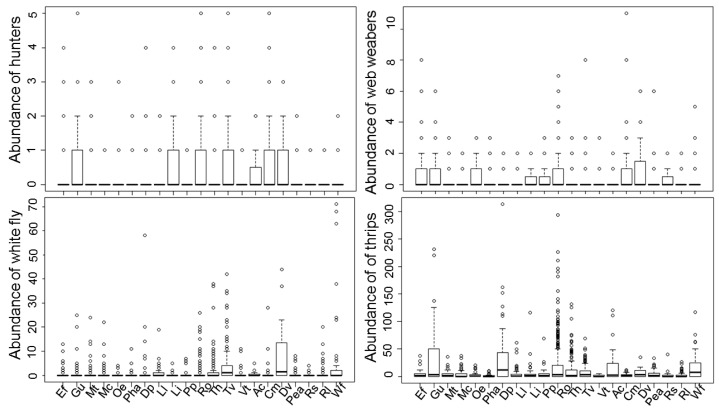
Abundance of hunter spiders, web-weaver spiders and the pests *Bemisia tabaci* and *Frankliniella occidentalis* in the different plant species using the plant codes from Table 1.

Spider abundance levels were higher in certain native plants (Table 3). Similarly, there were seasonal differences in the distribution of spiders (hunters and web-weavers) over time (Table 3). The highest level of pests was recorded outside when maximum temperatures were 31.3 °C and 20.9 °C for whitefly and thrips, respectively. Hunters were more abundant when maximum was 35.8 °C whereas web-weavers were abundant at 27.4 °C. Overall, the abundance of spiders was higher in a group of eleven plant species, consisting of *A. cytisoides*, *C. martimum*, *D. viscosa*, *E. fragilis*, *G. umbellata*, *L. intricatum*, *O. europaea*, *P. purpurea*, *R. sphaerocarpa*, *R. officinalis* and *T. vulgaris*, than in the other plant species (Figure 1). In particular, two native plants, *A. cytisoides* and *T. vulgaris*, supported a larger number of hunters, while web-weavers were more abundant in *E. fragilis*, *O. europaea* and *R. sphaerocarpa* (Figure 1)*.* Only five plant species, *G. umbellata*, *R. officinalis*, *T. vulgaris*, *A. cytisoides* and *D. viscosa*, showed a high occurrence of both spiders and pests (Figure 1). The results generated by the models used indicate that the abundance of *B. tabaci* and *F. occidentalis* had a significant effect on the occurrence of hunters (Table 3). Similarly, the abundance of *B. tabaci*, but not *F. occidentalis*, had a significant impact on web-weavers (Table 3).

### 3.2. Redundancy Analysis of Spiders, Pests and Other NEs in Native Plants

**Figure 2:**
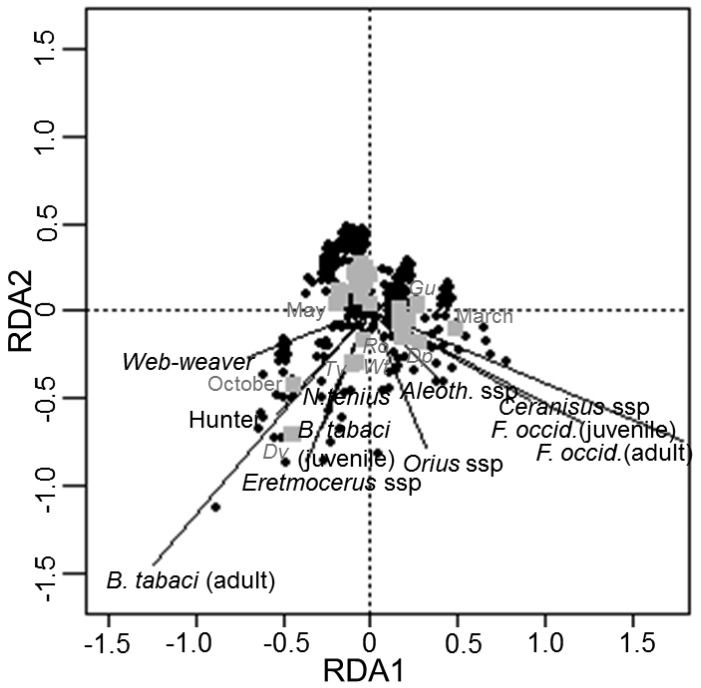
Redundancy Analysis (RDA) ordination diagram showing the variation in the abundance of the spider guilds, the pests *Bemisia tabaci* and *Frankliniella occidentalis* and their NEs (―) with respect to two nominal variables: native plants and sampling period in months (■). Native plants are labelled using plant code from Table 1.

The relationship between spiders, pests and other NEs in native plants was examined using RDA (Figure 2). RDA numerical output showed that all the explanatory variables accounted for 17% of the variation in arthropod data. Of this 17%, the first two axes accounted for 57% of the variation, with the first axis alone accounting for 33%. Adjusted R^2 was 15.6%, suggesting that other important data structures were not captured by the model. The RDA correlation triplot showed that *B. tabaci*, *N. tenuis*, *Eretmocerus* spp., as well as web-weaver and hunter spiders are situated to the left of the origin, indicating that all species correlate with one another. The abundance levels of these species were found to be highest on the plants, *D. viscosa*, *T. vulgaris* and *W. frutescens*, in the months of October and May. Specifically, *Eretmocerus* spp. and the predatory bug *N. tenuis* closely correlated with the nymphal stages of the pest. Adult-stage whiteflies correlated closely with hunter spiders and with web-weavers with intermediate values. With regard to *F. occidentalis*, the pest and its NEs are situated on the right in the diagram, which indicates that the abundance of spiders (hunters and web-weavers) does not correlate with either *F. occidentalis* or its NEs. Pest and NEs were more abundant in *D. pentaphyllum*, *R. officinalis* and *G. umbellata* in March. The parasitoid *Ceranisus* spp. closely correlated with the nymphal stages of the pest, whereas the predatory bug *Orius* spp., showed a weak positive correlation with the adult stages of *F. occidentalis*. *Aeolothrips* spp. correlated with adult *F. occidentalis* with intermediate values.

## 4. Discussion

**Figure 3:**
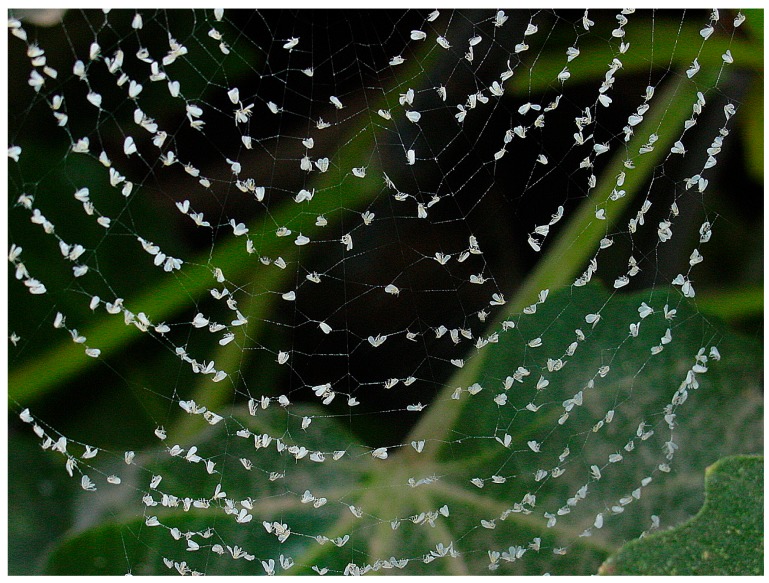
Example of *B. tabaci* adults trapped in webs built by web-weavers from crops surrounding greenhouses in the study area (Almeria, SE Spain). Photo: Jan van der Blom.

The aim of this study was to contrast spider abundance in different native shrubs, and the abundance of major pest species and specific biological agents, with a view to using these plants as habitat manipulation resources in Mediterranean greenhouses landscapes. Given the important role usually played by spiders in predatory arthropod communities in agroecosystems, we focused on the role of these plant species to assist spiders and improve BC outdoors. The spider communities collected from native plants belonged to a diverse range of families, although the orb-weaver *Neoscona subfusca*, as well as hunter spiders, accounted for over 89% of the spiders captured. The composition of spiders found in the area studied (SE Spain) is in line with studies of agroecosystems in Europe and the USA, where hunters were found to be more complex in terms of species composition, while web-weavers are usually highly uniform, with a numerical predominance of one family/species in many locations [26,43]. The spider assemblage found in native plants could potentially constitute part of an outdoor pest control strategy in Mediterranean greenhouse areas. For instance, philodromids, which prey on a wide variety of pests in fruit orchards, have been shown to be potential biocontrol agents [44], while crab spiders have been reported to be whitefly predators in cotton [33]. Specifically, certain hunter species captured in this study are also important NEs of *B. tabaci;* for example, *Thyene imperalis* in cotton crops [32]. Spiderlings of the crab spider *Xysticus kochi* may be effective predators of *F. occidentalis* in greenhouse pepper crops [37]. The biocontrol potential of small spiders such as web-weavers is generally limited due to low feeding frequency; however, these spiders, capable of building up large populations, could play an important ecological role reducing and stabilizing prey densities [35]. Moreover, webs, which increase the mortality of certain pests, have additional benefits in terms of BC, although these pests are not necessarily consumed by spiders [45,46]. Indeed, adult-stage *B. tabaci* are often trapped in webs built by web-weavers in crops in surrounding greenhouses (Figure 3). The *B. tabaci* remains have been identified and quantified in the gut of the native *Neoscona* species in cotton fields [36]. 

Month of sampling was such a highly significant variable in arthropod populations. Temperatures from late summer create the ideal conditions for whitefly to thrive in native vegetation outdoors. On the other hand, western flower thrips populations build up on native plants throughout the colder season, from December to May. The increase in spider populations was tied to the weather conditions from late-spring and summer, especially for hunter spiders, that remained abundant during summer (data not shown). In addition, it is important to note that hunters and web-weavers are more abundant in certain plant species. This information is crucial in ecological terms because it could provide clues as to where and when spiders are likely to be prevalent, indicating co-occurrence between spiders and horticultural pests and thereby show the likelihood of biocontrol. For instance, spiders were mainly found on the small Mediterranean shrub *Crithmum maritimum*. However, this plant, like other native shrubs such as *E. fragilis* or *L. intricatum*, supports very low levels of the two pests, *B. tabaci* and *F. occidentalis* (Figure 1), suggesting that spatial co-occurrence between pests and spiders might not occur in certain plant species. Nevertheless, some plants such as *G. umbellata*, *R. officinalis*, *D. viscosa*, *T. vulgaris* and *A. cytisoides* were selected by spiders and also supported levels of pests, suggesting that there is spatial co-occurrence between NEs and pests and therefore the probability of biocontrol. In fact, the GAM showed the significant effect of whiteflies on spider’s abundance (hunter and web-weavers) and the effect of *F. occidentalis* on hunter’s abundance. Some Thomisidae species have been reported to be potential predators of *F. occidentalis* in vegetable crops [37]. However, RDA showed no correlation between *F. occidentalis* and spiders, suggesting that spiders avoid preying on *F. occidentalis*. Indeed, thrips are rarely found to be part of spider food webs [34,35]. Therefore, the result that the GAM showed that *F. occidentalis* has a significant effect on hunters is in contrast to the RDA analysis. Adjusted R^2 was 15.6%. Therefore, a few other variables not captured by the model could be expected to be predictors of pest populations outdoors. Such factors could be different farming practices and pests’ abundance into greenhouses. The lack of correlation between *F. occidentalis* and spiders could therefore be partly explained by the different seasonal distribution patterns of spiders in native plants as compared to those of *F. occidentalis*, which is especially abundant outdoors during the colder months, especially in March. In fact, RDA showed that the abundance levels of *F. occidentalis* and its NEs were high in March, while spiders were more abundant during the warmer season. Therefore, it is possible that spiders and thrips co-occur for biocontrol only in a short period of time. Regarding the whitefly, hunters exhibit a strong positive relationship with adult and nymphal-stage *B. tabaci*, which points to the sessile juveniles of the pest as a suitable part of the spiders’ diet. The intermediate relationship reported between web-weavers and the two stages of whitefly could be explained by the prey suitability of *B. tabaci* for this spider guild, suggesting that web-weavers might prefer to feed on adult-stage rather than nymphal stage of the pest. Most web-weavers largely depend on relatively few prey groups available in large numbers in a particular environment, whereas hunters feed on both moving and sessile prey as a result of their mobile foraging strategy [34]. Regarding the NEs, the positive relationship between spiders (hunters and web-weavers) and whitefly NEs may also indicate consumption of the predatory bug *N. tenuis* or the parasitized nymphal-stage by spiders and thus unidirectional intraguild predation (spiders on whitefly NEs). For instance, web-weavers are known to mainly capture minute soft-bodied insects, with Heteroptera appearing to be a more suitable source of food for hunters [34]. Similarly, it has been found that generalist predators, such as carabid and staphylinid beetles, as well as linyphiid spiders, may disrupt parasitoid aphid control through direct and coincidental intraguild predation [47]. Nevertheless, the interaction between spiders and whitefly NEs supports the hypothesis of positive NE–whitefly interactions rather than intraguild predation between spiders and whitefly NEs. In fact, RDA showed that pest availability is a strong predictor of the abundance of NEs in native plants.

Finally, this study has shown that certain native plants, such us *A. cytisoides*, *C. martimum*, *D. viscosa*, *E. fragilis*, *G. umbellata*, *L. intricatum*, *O. europaea*, *P. purpurea*, *R. officinalis*, *R. sphaerocarpa* and *T. vulgaris*, may be especially attractive for hosting spiders. The density and diversity of plant-dwelling spiders appears to be closely tied to the architectural variations in the vegetation [48]. Plants are often important for spiders as sites for building webs, for sheltering against desiccation or natural enemies, for foraging, and for mating and oviposition [48,49,50,51]. Therefore, richness and composition of spider species on plants are influenced by the structural complexity and diversity of these plants. Vegetation with sufficient interspaces, greater height, lower leaf and branch densities favour the establishment of web weavers [48,49,50]. This was the case with, for instance, *G. umbellata*, *E. fragilis*, *O. europaea* and *R. sphaerocarpa*, which supported a higher abundance of web-weavers. On the other hand, low and sparse plants support a lower spider population. Exception to this rule in this study occurred in some low plants with high density of spiders, particularly hunters, like *C. maritimum* and *T. vulgaris*. This, however, could be explained by favourable microclimate or by presence of inflorescences (i.e., prey availability). It is known that thomisid spiders select plant-determined microhabitat (i.e., size and shape of leaves); and inflorescences can attract more guild spiders, including hunters, than vegetative branches [52]. Some plants of this study such as *G. umbellata*, *R. officinalis*, *D. viscosa*, *T. vulgaris* and *A. cytisoides* hosted spiders and pests. These five plants have already been identified for harboring pests and specific NEs populations’ outdoors [22]. Therefore, inclusion of plants species supporting spiders in hedgerows between greenhouses should optimize natural pest control whilst maintaining conservation value. By benefiting spiders, they will predate more whiteflies and thrips, and this will reduce their immigration into the greenhouses and, therefore, viruses transmitted by pests into crops. Since viruses that are transmitted by whitefly and western flower thrips tend to be found at lower densities in native flora than in crops [17], these plantations around greenhouses may act as phytosanitary barriers for pests and diseases that affect greenhouse horticultural crops. In agroecosystems based on annual crops, several non-host plants have been tested as barrier crops or intercrops to reduce whitefly colonization and virus transmission among main crops [53,54,55]. As the study was conducted with different experimentally planted shrub species, further field research using these candidate plants as plantations in the vicinity of greenhouses is required to confirm these results.

## 5. Conclusions

A group of 11 native shrub species, *A. cytisoides*, *C. martimum*, *D. viscosa*, *E. fragilis*, *G. umbellata*, *L. intricatum*, *O. europaea*, *P. purpurea*, *R. officinalis*, *R. sphaerocarpa* and *T. vulgaris*, was found to support a large number of spiders. Specifically, *E. fragilis*, *O. europaea* and *R. sphaerocarpa* supported a larger number of web-weavers, while *T. vulgaris* and *A. cytisoides* supported more hunters. Only five of them, *G. umbellata*, *R. officinalis*, *D. viscosa*, *T. vulgaris* and *A. cytisoides*, hosted higher abundance of spiders and pests. Abundance of both pests had a significant effect on the abundance of hunter spiders, while the abundance of *B. tabaci*, unlike *F. occidentalis*, affected the abundance of web-weavers. Results also suggest that spiders can offer a complementary niche: web-weavers may play an important role as adult-stage predators of whitefly, whereas hunters may play as predators of nymphal stages of the pest. Nevertheless, it is important to highlight the positive correlation detected between spiders and whitefly NEs. This suggests that the predatory bug *N. tenuis* and immobile stages of whiteflies (parasitized or not by *Eretmocerus* spp.) could be potential prey for spiders, a hypothesis which is worthy of further study in the future. In conclusion, the results show that there is a positive relationship between spider and pest abundance on some plants, suggesting potential for biological control due to spatial and temporal occurrence. This relationship was higher for *Bemisia tabaci* than for *Frankliniella occidentalis*. On the basis of these results, future field trials are needed to see whether conservation strategies involving the establishment of native plants suitable for spiders outdoors may provide changes or reduction on pest populations, especially those of *B. tabaci*.

## Figures and Tables

**Table 1:** Native shrub species selected for habitat management in Mediterranean greenhouse areas.

Species Assayed	Common Name	Family	Plant Code	Number Assayed
*Ephedra fragilis* Desf.	joint pine	Ephedraceae	Ef	7
*Genista umbellata* Poir.	bolina	Fabaceae	Gu	7
*Macrochloa tenacissima* (L.) Kunth	alfa grass	Poaceae	Mt	9
*Myrtus communis* L.	myrtle	Myrtaceae	Mc	7
*Olea europaea* var*. sylvestris* L.	wild olive tree	Oleaceae	Oe	3
*Phillyrea angustifolia* L.	false olive	Oleaceae	Pha	10
*Dorycnium pentaphyllum* Scop.	prostrate canary clover	Fabaceae	Dp	6
*Lavandula latifolia* Medik.	spike lavender	Lamiaceae	Li	6
*Lycium intricatum* Boiss.	cambrón	Solanaceae	Li	4
*Phlomis purpurea* L.	purple phlomis	Lamiaceae	Pp	2
*Rosmarinus officinalis* L.	rosemary	Lamiaceae	Ro	25
*Thymus hyemalis* Lange.	winter thyme	Lamiaceae	Th	17
*Thymus vulgaris* L.	thyme	Lamiaceae	Tv	19
*Viburnum tinus* L.	laurustinus	Adoxaceae	Vt	4
*Anthyllis cytisoides* L.	albaida	Fabaceae	Ac	2
*Crithmum maritimum* L.	rock samphire	Apiaceae	Cm	6
*Dittrichia viscosa* (L.) Greuter	false yellowhead	Asteraceae	Dv	2
*Periploca angustifolia* Labill.	cornical	Asclepiadaceae	Pea	6
*Retama sphaerocarpa* (L.) Boiss.	yellow broom	Fabaceae	Rs	3
*Rhamnus lycioides* subsp*. lycioides* L.	Mediterranean buckthorn	Rhamnaceae	Rl	10
*Whitania frutescens* (L.) Pauquy.	oroval	Solanaceae	Wf	6

**Table 2:** Frequency (%) of spiders collected in 21 newly planted shrubs established in a highly disturbed horticultural system.

Taxa	Frequency (%)
Web-weavers	50.6
Juveniles	13.1
Araneidae [*Neoscona subfusca*]	78.7
Araneidae (sp. 1 sp. 2 sp. 3)	4.7
Theridiidae [*Anelosimus aulicus*]	1.5
Linyphiidae/Tetragnatidae/Pholcidae	2.0
Hunting spiders	49.4
Salticidae [*Thyene imperialis*]	23.2
Salticidae [*Heliophanus aeneus*]	13.7
Thomisidae [*Xysticus kochi*]	20.8
Thomisidae [*Thomisus onustus*]	4.7
Thomisidae [*Xysticus bufo*]	0.6
Oxyopidae [*Oxyopes* spp*.*]	10.0
Oxyopidae [*Peucetia viridans*]	0.5
Philodromidae [*Pulchellodromus* spp.]	21.1
Philodromidae [*Philodromus dispar*]	4.5
Liocranidae/Lycosidae	0.9

**Table 3:** Results of the Generalized Additive Model (GAM) analysing the abundance of spider guilds in 21 native shrubs with different abundance of pests and sampling period as covariates.

Model	Types of Variables	Variables	Estimate	SE	χ^2^	df	*p* Value
Hunter spiders	Numerical	Whitefly abundance	0.015	0.005	18.771	1	<0.001
	Numerical	Thrips abundance	−0.005	0.004	7.943	1	<0.01
	Categorical	Plant species			193.792	20	<0.001
	Smoothed	Month of sampling			113.1	8.35	<0.001
Web-weavers	Numerical	Whitefly abundance	0.014	0.007	4.0544	1	<0.05
	Numerical	Thrips abundance	−0.004	0.004	0.782	1	0.3765
	Categorical	Plant species			164.204	20	<0.001
	Smoothed	Month of sampling			336	8.89	<0.001
